# Advancing LED technology: the FDCSP element’s breakthrough in mini and micro-LED packaging and backlight module enhancement

**DOI:** 10.1186/s11671-024-04033-5

**Published:** 2024-05-28

**Authors:** Jo-Hsiang Chen, Che-Hsuan Huang, Tzu-Yi Lee, Fang-Chung Chen, Tsung-Sheng Kao, Hao-Chung Kuo

**Affiliations:** 1 Department of Photonics, College of Electrical and Computer Engineering, National Yang Ming Chiao Tung University, Hsinchu, 30010 Taiwan; 2 Semiconductor Research Center, Hon Hai Research Institute, Taipei, 11492 Taiwan

**Keywords:** Freeform-design, Chip scale package, Energy conservation, Light source calculation, Mini LED, Light emitting diode

## Abstract

In this research, we introduce an advanced methodology for the calculation of bulk light sources tailored for free-form surface design, focusing on the principle of energy conservation. This method is especially relevant for the evolving needs of micro-LED packaging, highlighting its potential in this burgeoning field. Our work includes the development of an algorithm for creating freeform-designed chip-scale package (FDCSP) components. These components seamlessly integrate LEDs and lenses, underscoring our commitment to advancing free-form surface design in chip-level packaging. By adhering to the principle of energy conservation, our approach facilitates a meticulous comparison of simulation outcomes with predefined target functions. This enables the iterative correction of discrepancies, employing layering techniques to refine the design until the simulated results closely align with our goals, as demonstrated by an appropriate difference curve. The practical application of these simulations leads to the innovative design of FDCSP devices. Notably, these devices are not just suitable for traditional applications in backlight modules but are explicitly optimized for the emerging sector of micro-LED packaging. Our successful demonstration of these FDCSP devices within backlight modules represents a significant achievement. It underscores the effectiveness of our design strategy and its expansive potential to transform micro-LED packaging solutions. This research not only contributes to the theoretical understanding of energy conservation in lighting design but also paves the way for groundbreaking applications in micro-LED and backlight module technologies.

## Introduction

In recent years, display technologies have rapidly evolved, with traditional LCD displays no longer meeting consumer demands. This has led to the emergence of new technologies like Mini-LED and Micro-LED displays [1–4]. The efficiency of light-emitting diodes (LEDs) has significantly improved due to advancements in the epitaxy process and semiconductor wafer process, enabling the use of LEDs in various fields such as panel backlighting and large-scale outdoor signboards [5]. Mini-LED and Micro-LED displays have gained industry attention for their high current density operation, high brightness, and high contrast. Mini-LED backlight technology, in particular, offers more local dimming zones and thinner design options compared to traditional edge-lit backlights [6,7]. The pursuit of fine pitch technologies in the display industry has been vigorous, with many researchers and companies dedicating efforts to Mini-LED and Micro-LED technologies due to their unique features. Mini-LEDs can match the total thickness of an edge-lit backlight while providing additional benefits like more local dimming zones, high brightness, and high contrast. As consumer demand for higher display quality increases, high dynamic range (HDR) has become a critical feature for next generation displays. HDR images offer a greater dynamic range and resolution, better reproducing natural scenes [8,9]. Achieving HDR typically requires high peak brightness, excellent dark state control, accurate grayscale, wide color gamut, and more [10]. Local dimming technology has been effective in suppressing dark states and enhancing contrast to achieve HDR. LCD backlight units with segmented LEDs allow for independent dimming of local areas to match the displayed image content. In direct backlight systems with local dimming, two types of LED packages are commonly used: traditional surface mount device (SMD) LEDs [11,12], and chip scale package (CSP) LEDs [8,13]. The SMD LED manufacturing process is simpler and easier for mass production [14]. However, the viewing angle of SMD LEDs is often limited by the cavity substrate, requiring an auxiliary lens over the LEDs [15]. Consequently, CSP LEDs, with their larger viewing angles, better light extraction efficiency, low cost, and easy assembly, have become a preferred package type in application modules, especially in LCD Backlight Unit (BLU) [16–18]. For BLU applications, the light source with a secondary lens is used in direct-lit backlights and mounted on the PCB board. However, as BLU designs continue to reduce backlight thickness, secondary optical lenses with freeform designs face spatial challenges [19,20]. In response to the miniaturization trend in BLU solutions and to minimize the loss of reflected light caused by the air gap between the light source and the secondary lens, the focus of optical design has shifted towards LEDs. Although formulas for free-form surfaces are quite mature, they are primarily based on point light sources [21]. This approach can yield good results when the lens to light source size ratio is large enough. However, when applied to BLU designs with miniaturized lenses, achieving the desired design results is often challenging. By integrating this principle, we propose the development of a novel freeform-designed chip-scale package (FDCSP) element structure. This approach not only addresses the specific needs of BLU designs but also extends its utility to the burgeoning field of micro-LED packaging, offering a versatile solution that enhances both form and function in miniature lighting applications. Our algorithm facilitates the design of the FDCSP element, incorporating optical design principles that are critical for the success of BLU designs and, by extension, for advancing micro-LED technology.

The development of the FDCSP element, tailored for BLU and micro-LED applications, represents a significant advancement in chip-scale packaging technology. It showcases our commitment to pushing the boundaries of lighting design, marrying the technical rigor of energy conservation calculations with the practical demands of modern, efficient, and compact lighting solutions. This approach not only promises improved outcomes for conventional BLU designs but also paves the way for innovative applications in the rapidly evolving micro-LED packaging sector, underscoring the potential of our method to revolutionize the design and functionality of next-generation lighting systems. In terms of system integration, advancements in photonic integration and high-speed optical communication systems have highlighted the significant role of semiconductor quantum dots in enhancing optoelectronic device performance [22]. Quantum dots possess excellent optical and electronic properties, providing precise spectral characteristics and high efficiency, suitable for compact optical modules. The latest developments in quantum dot laser technology, including precise wavelength control, make them crucial components in future high-speed optical communication systems [23]. Additionally, quantum dot technology combined with Micro-LEDs forms a unique CSP structure that improves optical efficiency, reduces costs, and enhances assembly convenience. This new CSP structure will bring revolutionary applications to the Micro-LED packaging field, revolutionizing the design and functionality of next-generation optoelectronic technology.

## Materials and methods

### Geometric-optical analysis

#### Traditional methodology

Due to the directional nature of light, its path can be accurately estimated using ray tracing calculations. Ray-tracing software, such as LightTools and Tracepro [24–26] has been developed to assist designers in addressing more complex challenges. One of the most advanced methods in optical design is freeform design. Various articles highlight the fundamental approach to designing a programming process that enhances efficiency and success in tackling larger-scale problems.

This basic method adheres to Snell’s Law and encompasses four key components: (1) definition of the target pattern, (2) geometrical boundary conditions, (3) the relative size between the light source and the optical component, and (4) the calculation using the reverse of Snell’s Law.

According to the statements above, all these processes can be encapsulated by formula (1). The application of the reverse Snell’s Law can be expressed as an operation⊛ (reverse Snell’s Law). The outcomes of freeform design can be represented by [freeform matrix], [Target image], and [Point source], respectively [27].

1 $$\left[ {{\text{freeform}}\;{\text{matrix}}} \right] = \left[ {{\text{Target}}\;{\text{image}}} \right]{ \circledast }\left( {{\text{reverse}}\;{\text{Snell}'} {\text{s}}\;{\text{law}}} \right)\left[ {{\text{Point}}\;{\text{source}}} \right]$$

### Revised freeform design rule

#### Concept of revised methodology

Although the reverse-Snell’s law method offers an efficient solution for executing optical designs, it encounters challenges when addressing near-field issues [28]. This is because the reverse-Snell’s law assumes that all rays originate from the same point, necessitating that the optical device’s geometrical size be significantly larger than the light source. In response to these limitations, this paper proposes a revised model to apply the freeform method to LED shape design, making the LEDs suitable for BLU applications. The model introduces a set of formulas, designated as formulas (2) to (4). Formula (2) outlines the process of ray tracing simulation, indicating that a light class is comprised of light rays after passing through a free-form surface, with the symbol “ ⊗ (ray trace)” denoting the ray tracing operation. However, formula (1), based on the reverse-Snell’s law, is applicable only to point light sources and struggles with the consideration of light source volume in the context of backlight module applications. Thus, to design a general free-form surface model for surface light sources, we introduce the method of objective function correction. This involves modifying the objective function to an effective target image, aiming to incorporate this effective target image and point source into formula (1). The resulting freeform matrix should enable the original target image to achieve the desired outcome after undergoing the ray tracing process defined in formula (2).

To enhance this method, we introduce the concept of energy conservation. A difference curve is generated by comparing the simulation results produced by formula (2) with the objective function at each iteration. Utilizing the principle of normalization of the total area, we adjust the energy increase and decrease for each group of positions. This correction forms an effective target image that meets the requirements of the new objective function.

2 $$\left[ {{\text{Image}}} \right] = \, \left[ {\text{freeform matrix}} \right] \otimes \left( {\text{ray trace}} \right)\left[ {\text{Light source}} \right]$$

3 $$\left[ {{\text{Target}}\;{\text{image}}} \right] = \left[ {{\text{Effective}}\;{\text{target}}\;{\text{image}}} \right]{ \circledast }\left( {{\text{reverse}} - {\text{Snell}'} {\text{s}}\;{\text{law}}} \right)\left[ {{\text{Point}}\;{\text{source}}} \right] \otimes \left( {{\text{ray}}\;{\text{trace}}} \right)\left[ {{\text{Light}}\;{\text{source}}} \right]$$

4 $$\left[ {{\text{Effective}}\;{\text{target}}\;{\text{image}}} \right] = \left[ {{\text{Energy}}\;{\text{conservation}}\;{\text{fix}}} \right]{ \oslash }\left[ {{\text{Target}}\;{\text{image}}} \right]$$

#### Design flow

**Fig. 1:**
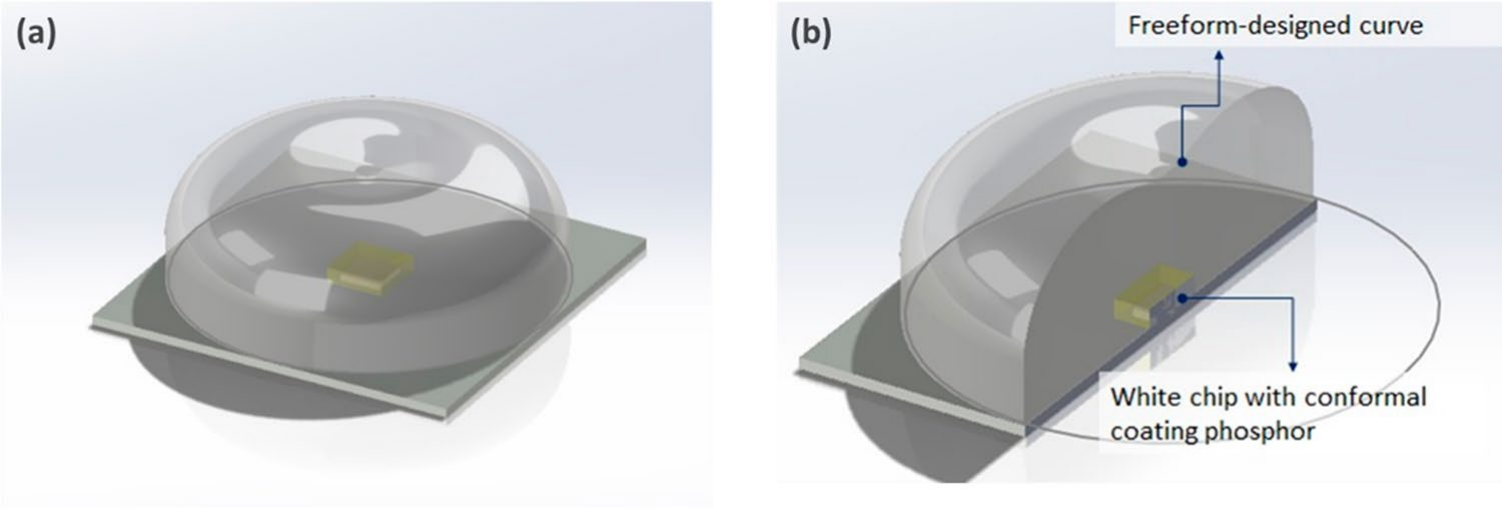
**a** Overall appearance. **b** Sectional view

This paper presents the FDCSP structure, designed to integrate LEDs and lenses effectively. The schematic diagrams of the FDCSP are depicted in Fig. 1a, b, illustrating the device’s two noteworthy characteristics. Firstly, a white CSP is centrally embedded on the substrate’s surface, ensuring a compact and efficient light source integration. Secondly, the device features a freeform-designed surface, optimizing light distribution and focusing. Together, these characteristics enable the FDCSP to function as an integrated device, combining the benefits of an LED with those of a secondary lens.

**Fig. 2:**
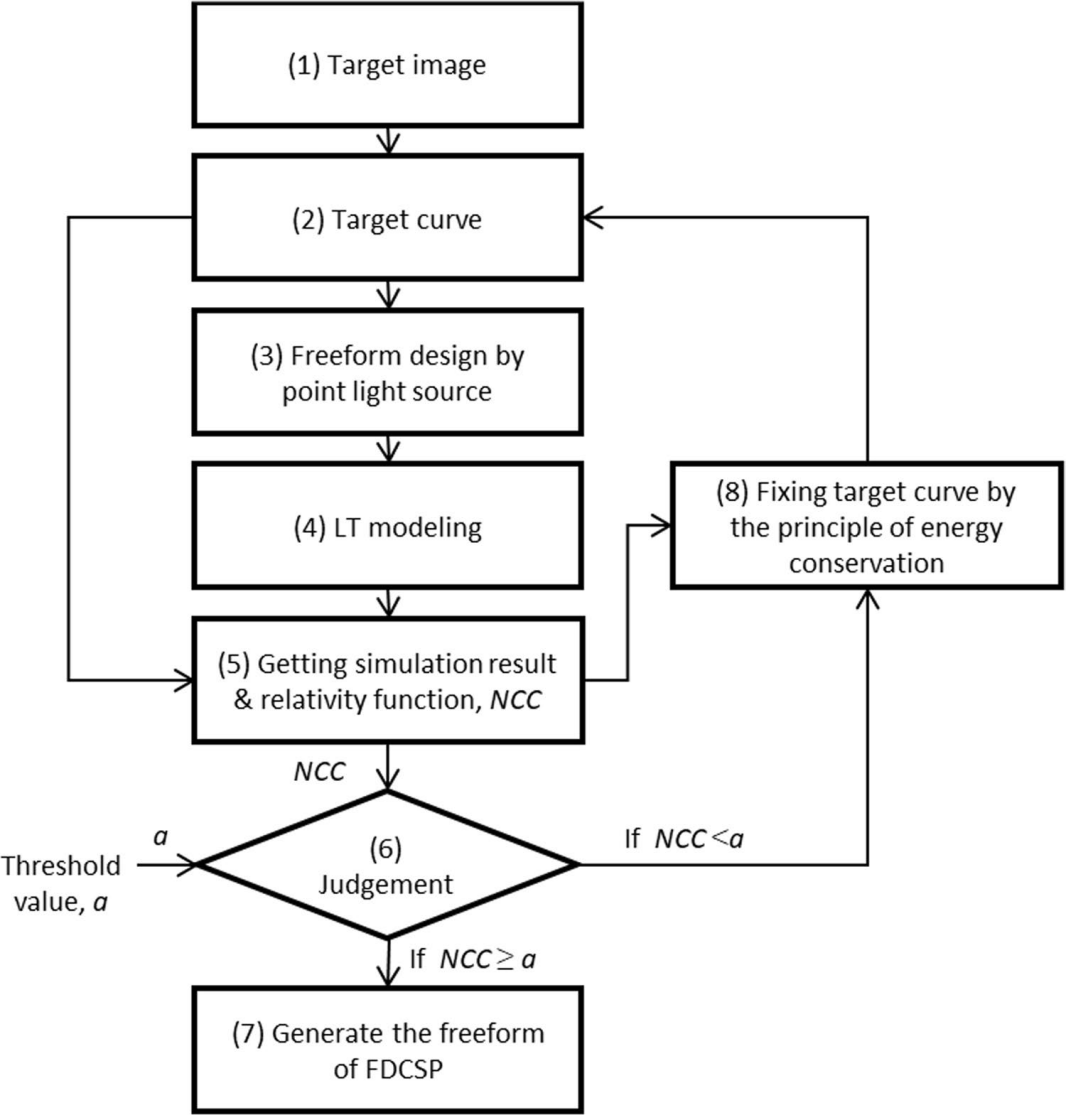
Design flow chart of FDCSP

The design flow chart for the FDCSP, depicted in Fig. 2, outlines the systematic approach undertaken in this project. Initialize the design process by setting the environmental and object parameters in LightTools, with the main parameters outlined in Table 1.

1. Derive the target function from the image to generate the initial freeform surface design.
2. Import the initial freeform surface into the LightTools model to commence ray tracing simulation.
3. Evaluate the simulation results by comparing them against the target function.
4. Apply the principle of energy conservation to formulate an effective target function.
5. Use the effective target function to create a revised freeform surface.
6. Repeat the process of importing the freeform surface into the LightTools model for ray tracing simula-tion (step 2), and then evaluate the simulation results by comparing them with the target function (step 3). During this iterative process, calculate the Normalized Correlation Coefficient (NCC) using formula 5, where Xmn and Ymn represent the values of the experimental and simulated data, respectively, and X and Y denote the mean values of the experimental and simulated data across the angular range. It’s essential to ensure that all simulation curves, across various Correlated Color Temperatures (CCT), achieve an NCC index of at least 99%.

**Table 1:** The mainly parameters on the LightTools.^8^

Light source	Freeform lens properties	Bottom reflectivity
White chip-scale package Amount of ray: 3,000,000 Data of ray source are measured by Source Imaging Goniometer (SIG)	Amount of freeform point: 10,000 Material’s refractive index: 1.51 Freeform surface: Fresnel loss	85% and Lambertian type of reflectivity Data is measured by OITC MCP-9800

This step is crucial for verifying the accuracy and reliability of the freeform surface design against the intended target function, ensuring that the design meets the high standards required for practical application.

5 $$NCC = \frac{{\mathop \sum \nolimits_{m} \mathop \sum \nolimits_{n} \left( {X_{mn} - \overline{X}} \right)\left( {Y_{mn} - \overline{Y}} \right)}}{{\sqrt {\left[ {\mathop \sum \nolimits_{m} \mathop \sum \nolimits_{n} \left( {X_{mn} - \overline{X}} \right)^{2} } \right]\left[ {\mathop \sum \nolimits_{m} \mathop \sum \nolimits_{n} \left( {Y_{mn} - \overline{Y}} \right)^{2} } \right]} }}$$

If the NCC is greater than or equal to the threshold value, proceed to step (7).

(7) Break the loop and complete the design flow.
  If the NCC does not meet the threshold value, move to step (8) for further adjustments.
(8) Repeat steps (4) to (6).

**Fig. 3:**
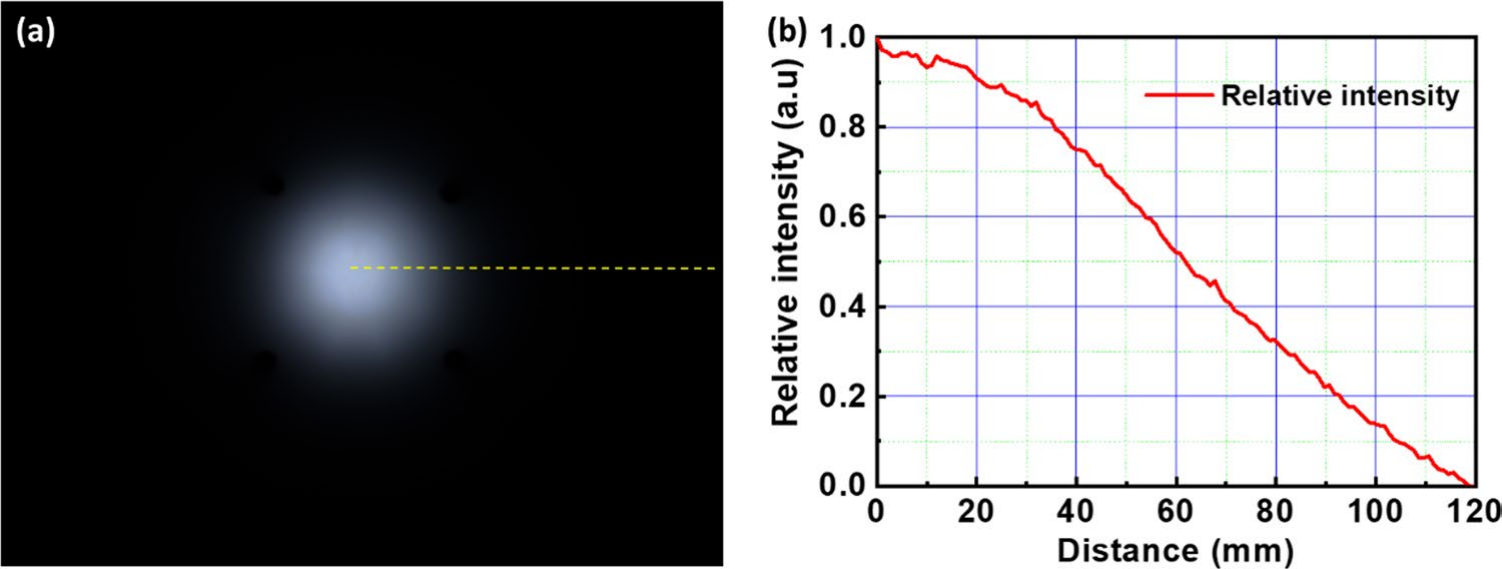
**a** The target image and **b** the target function which is accessed from the target image

Figure 3a displays an image of a single sample captured by a CCD camera on the module. Image processing techniques are then employed to calculate the light intensity profile of the spot, as shown in Fig. 3b. This profile is subsequently utilized to further refine and continue the algorithm design. This revision aims to succinctly describe the methodological steps taken from capturing an image of the sample to analyzing its light intensity profile for use in ongoing algorithm development.

## Result and discussion

### Simulation result

**Fig. 4:**
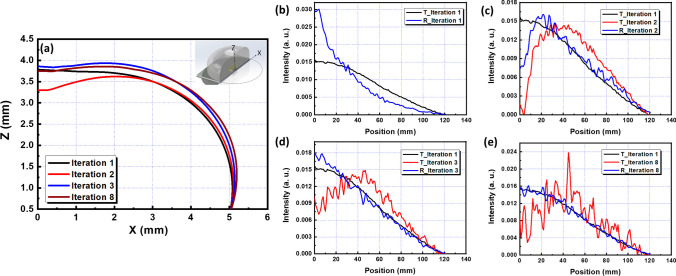
**a** Freeform design curve. **b** Freeform result of 1st iteration. **c** Freeform result of 2nd iteration. **d** Freeform result of 3rd iteration. **e** Freeform result of 8th iteration

Following the design flow outlined in Fig. 2, simulations were conducted for the design of a BLU application. Figure 4a presents curves that represent the surface designed by a point light source for the objective function. On these curves, the X-axis denotes the distance from the center of the element along the XY plane, while Z indicates the direction perpendicular to the element and the XY plane. These curves shape into the free area surface of the element, illustrating the FDCSP design process. Each curve depicts the design results of the free area at different iteration times. Figure 4b compares the results between the point light source and the free area surface designed by the original objective function after simulation in LightTools. ‘T_Iteration 1’ refers to the objective function for the initial free-form surface design, and ‘R_Iteration 1’ represents the outcome of the first free-form surface operation in LightTools. The comparison reveals that ‘R_Iteration 1’ exhibits a smaller full width at half maximum (FWHM) than ‘T_Iteration 1’, with energy more concentrated at the center (near x = 0), enhancing central brightness. The Normalized Correlation Coefficient (NCC) value for this comparison is 0.887. After analyzing the differences between ‘R_Iteration 1’ and ‘T_Iteration 1’, the objective function is refined to ‘T_Iteration 2’, as depicted in Fig. 4c. This adjustment reduces the energy distribution at x < 20 and increases the energy target at x > 40, while maintaining the total energy level. The result, ‘R_Iteration 2’, shows a lower energy distribution at x < 20 compared to ‘T_Iteration 1’, with an improved NCC value of 0.92. The process continues to the third iteration, shown in Fig. 4d, where ‘R_Iteration 3’ closely matches the profile of ‘T_Iteration 1’, achieving an NCC of 0.989. Through continuous iterations, as illustrated in Fig. 4e, the optimized objective function is achieved by the 8th iteration. ‘T_Iteration 8’ is designed to accomplish the free-form surface objectives set in ‘T_Iteration 1’, with the design results pushing the NCC to 0.995.

**Fig. 5:**
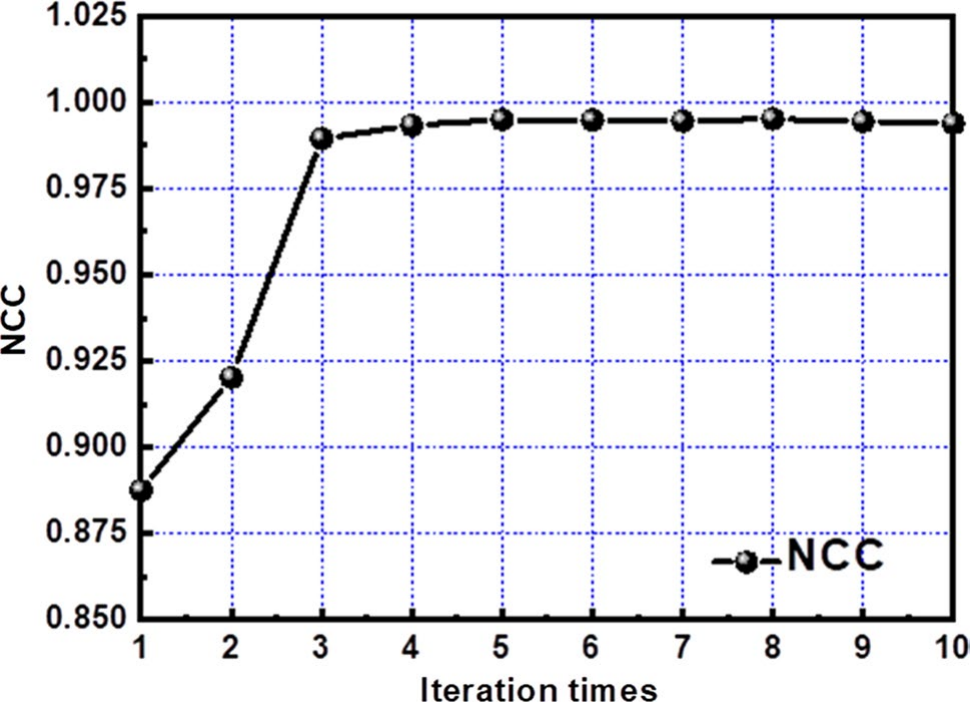
NCC result of different iteration times

Figure 5 illustrates the configuration of the simulated data, with the experimental findings indicating that the NCC value surpasses the 0.99 threshold after just four iterations. To verify the stability of the NCC value as a consistent solution, the test was extended from iteration 5 to iteration 10. The results demonstrated remarkable stability, confirming that a stable solution could indeed be achieved within just four iterations. As detailed in Table 2, the NCC values between iteration 4 and iteration 10 consistently remained above 0.993, reaching an optimal NCC of 0.995 in iteration 8.

**Table 2:** NCC values for successive iterations

Iteration	1	2	3	4	5	6	7	8	9	10
NCC	0.887	0.920	0.989	0.993	0.994	0.994	0.994	0.995	0.994	0.993

### Virtual experiment result

**Fig. 6:**
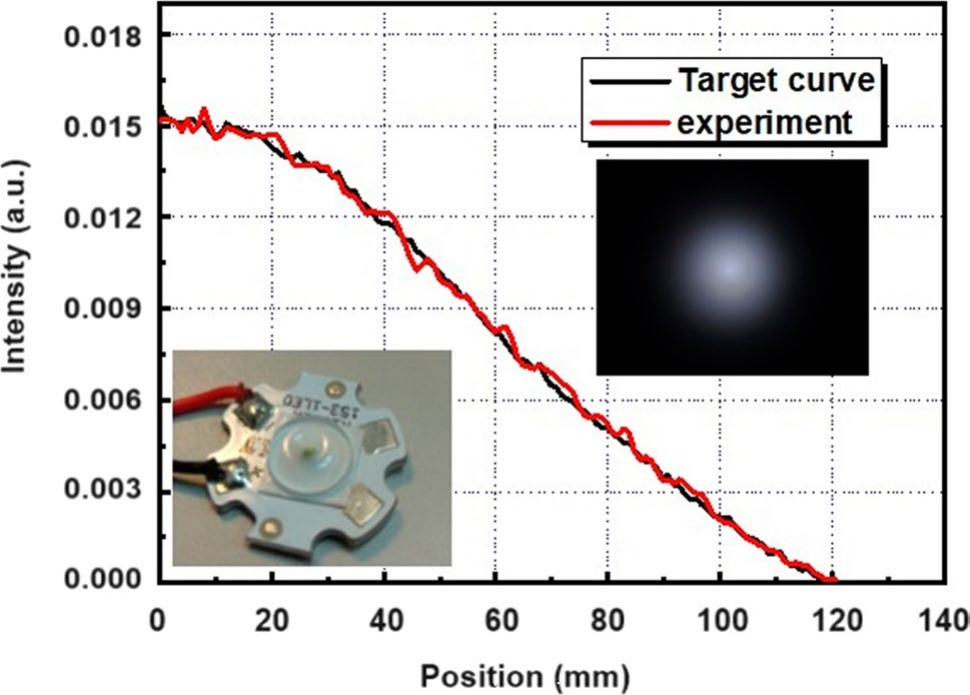
Finished product performance

Based on the simulation results depicted in Fig. 5, a physical sample was fabricated, and its performance within a backlight module was measured and compared against the objective function, as illustrated in Fig. 6. The comparison reveals a high degree of overlap between the actual sample’s performance and the targeted results. This indicates that the FDCSP, designed and manufactured through the energy-conserving free-form surface iteration method, successfully meets the specific light pattern requirements necessary for backlight module applications.

## Conclusions

This study introduces an innovative FDCSP element, which leverages the principles of volume miniaturization and integrated LED-lens molding. Our approach synthesizes methodologies from wafer-level chip-scale packaging with freeform surface design, significantly enhancing the utility of this design for micro-LED packaging applications. The FDCSP’s architecture allows it to perform primary optical functions while also showcasing secondary optical characteristics, offering a comprehensive solution for advanced lighting applications. Utilizing the principle of energy conservation, we have devised a novel design method that initially corrects for the volume light source on the free-form surface, transitioning from a simplistic point light source model. This adjustment process, remarkably efficient, reached an NCC value above the 0.99 threshold within just four iterations, highlighting the precision of our design. Through detailed simulation, we have crafted FDCSP components that are not only suitable but optimized for backlight module applications, extending their potential to micro-LED packaging solutions. The successful production and testing of physical samples affirm their high efficacy and validate their application in enhancing backlight modules, marking a significant step forward in the field of advanced LED packaging and design.

## References

[1] Liu Z (2020). Micro-light-emitting diodes with quantum dots in display technology. Light Sci Appl.

[2] Lee T-Y (2022). Technology and applications of micro-LEDs: their characteristics, fabrication, advancement, and challenges. ACS Photon.

[3] Miao WC (2024). Microdisplays: mini-LED, micro-OLED, and micro-LED. Adv Opt Mater.

[4] Wu T (2018). Mini-LED and micro-LED: promising candidates for the next generation display technology. Appl Sci.

[5] Nakamura S, Krames MR (2013). History of gallium–nitride-based light-emitting diodes for illumination. Proc IEEE.

[6] Tan G, Huang Y, Li M-C, Lee S-L, Wu S-T (2018). High dynamic range liquid crystal displays with a mini-LED backlight. Opt Express.

[7] Lin C-H (2019). Hybrid-type white LEDs based on inorganic halide perovskite QDs: candidates for wide color gamut display backlights. Photon Res.

[8] Huang C-H (2019). Ultra-high light extraction efficiency and ultra-thin mini-LED solution by freeform surface chip scale package array. Crystals.

[9] Chen H, Zhu R, Li M-C, Lee S-L, Wu S-T (2017). Pixel-by-pixel local dimming for high-dynamic-range liquid crystal displays. Opt Express.

[10] Krause M, Riplinger M, Louis AK, Xu C (2018). A new class of very efficient algorithms for local dimming. Optim Eng.

[11] Raypah ME, Sodipo BK, Devarajan M, Sulaiman F (2015). Estimation of optical power and heat-dissipation factor of low-power SMD LED as a function of injection current and ambient temperature. IEEE Trans Electron Devices.

[12] Kuo C-FJ, Fang T-Y, Lee C-L, Wu H-C (2019). Automated optical inspection system for surface mount device light emitting diodes. J Intell Manuf.

[13] Fan J, Yu C, Qian C, Fan X, Zhang G (2017). Thermal/luminescence characterization and degradation mechanism analysis on phosphor-converted white LED chip scale packages. Microelectron Reliab.

[14] Scott SM, Ali Z (2021). Fabrication methods for microfluidic devices: an overview. Micromachines.

[15] Luo X, Wu D, Wang K, Liu S (2017). Freeform optics for LED packages and applications.

[16] Lai C-F, Tien Y-C, Tong H-C, Zhong C-Z, Lee Y-C (2018). High-performance quantum dot light-emitting diodes using chip-scale package structures with high reliability and wide color gamut for backlight displays. RSC Adv.

[17] Virey E (2018). Are microLEDs really the next display revolution?. Inf Display.

[18] Yu C-S, Lin C-S (2010). Building TQM by integrated strategies for B2B industry: next-generation lighting technology in Taiwan. Total Qual Manag.

[19] Leiner C, Nemitz W, Schweitzer S, Wenzl FP, Sommer C (2020). Design procedure for ultra-thin free-form micro-optical elements allowing for large DHR values and uniform irradiance distributions of ultrathin direct-lit luminaires. OSA Continuum.

[20] Chen F; Kuo, J. In-vehicle display technology. In: Advanced Driver Assistance Systems and Autonomous Vehicles: From Fundamentals to Applications. Springer Nature Singapore. 2022. 339–419.

[21] Brix K, Hafizogullari Y, Platen A (2015). Designing illumination lenses and mirrors by the numerical solution of Monge–Ampère equations. JOSA A.

[22] Chen E (2024). Broadband beam collimation metasurface for full-color micro-LED displays. Opt Express.

[23] Chen E, et al. Collimated LED array with mushroom-cap encapsulation for near-eye display projection engine. IEEE J Select Top Quant Electron. 2024;30:2000410.

[24] Jiao F, Liu L, Cheng W, Li C, Zhang X (2022). Review of optical measurement techniques for measuring three-dimensional topography of inner-wall-shaped parts. Measurement.

[25] Dey R, Bordatchev EV, Tauhiduzzaman M, Reshef H. In: Physics, simulation, and photonic engineering of photovoltaic devices. SPIE. 2012;8256:368–376.

[26] Park S, Shin Y, Oh K, Bang T (2016). Optical characteristics of LGP depending on the scattering pattern orientation for flat-type LED lighting. Opt Rev.

[27] Wu R (2018). Design of freeform illumination optics. Laser Photon Rev.

[28] Wee WH. The physics of negative refraction and transformation optics (Doctoral dissertation, Department of Physics, Imperial College London); 2011.

